# Immune activation and exhaustion marker expression on T-cell subsets in ART-treated adolescents and young adults with perinatal HIV-1 infection as correlates of viral persistence

**DOI:** 10.3389/fimmu.2023.1007626

**Published:** 2023-03-23

**Authors:** Yuyang Huang, Adit Dhummakupt, Priya Khetan, Tricia Nilles, Weiqiang Zhou, Prakriti Mudvari, Joseph Szewczyk, Ya Hui Chen, Eli Boritz, Hongkai Ji, Allison Agwu, Deborah Persaud

**Affiliations:** ^1^ Bloomberg School of Public Health, Johns Hopkins University, Baltimore, MD, United States; ^2^ Department of Pediatric Infectious Disease, Johns Hopkins University School of Medicine, Baltimore, MD, United States; ^3^ Vaccine Research Center, National Institute of Allergy and Infectious Diseases, National Institute of Health, Bethesda, MD, United States

**Keywords:** HIV - human immunodeficiency virus, activation, exhaustion, correlates and predictors, perinatally acquired HIV

## Abstract

HIV-1 infection in memory CD4+ T cells forms a latent reservoir that is a barrier to cure. Identification of immune biomarkers that correlate with HIV-1 reservoir size may aid with evaluating efficacy of HIV-1 eradication strategies, towards ART-free remission and cure. In adults living with non-perinatal HIV-1, the immune exhaustion marker PD-1 on central memory CD4+ T cells (Tcm) correlates with measures of HIV-1 reservoir size. Immune correlates of HIV-1 are less defined in adolescents and young adults with perinatal HIV-1. With multi-parameter flow cytometry, we examined immune activation (CD69, CD25, HLA-DR), and exhaustion (PD-1, TIGIT, TIM-3 and LAG-3) markers on CD4+ T cell subsets (naïve (Tn), central memory (Tcm), and the combination (Ttem) of transitional (Ttm) and effector memory (Tem) cells, in 10 adolescents and young adults living with perinatal HIV-1 (median age 15.9 years; median duration of virologic suppression 7.0 years), in whom HIV-1 reservoir size was measured with the Intact Proviral HIV-1 DNA Assay (IPDA) and an enhanced Tat/Rev limiting dilution assay (TILDA). RNA-seq was also performed on the unstimulated CD4+ T cells. The median total HIV-1 DNA concentration in memory CD4+ T cells was 211.90 copies per million CD4+ T cells. In the 7 participants with subtype B HIV-1 infection, the median intact proviral DNA load was 7.96 copies per million CD4+ T cells. Levels of HLA-DR and TIGIT on the Ttem were correlated with total HIV-1 DNA (r=0.76, p=0.015) and (r=0.72, p=0.023), respectively, but not with intact proviral load or induced reservoir size. HIV-1 DNA load was also positively correlated with transcriptional clusters associated with HLA-DR expression by RNA-seq. In contrast, PD-1 expression on Tcm was inversely correlated with total HIV-1 DNA (r=-0.67, p=0.039). Reservoir size by IPDA and TILDA were correlated (r=0.81, p=0.036). Thus, in this cohort of youths with long-standing treated perinatal infection, HLA-DR and TIGIT on Ttem were the key correlates of HIV-1 infected cell frequencies by total HIV-1 DNA, and not PD-1. Total HIV-1 DNA was negatively correlated with PD-1 expressing Tcm. These differences in longstanding perinatal HIV-1 infection compared with adult infection requires further study in larger cohorts.

## 1 Introduction

Globally, 1.7 million children under the age of 15 are living with HIV-1 with an estimated 160,000 new infections in 2021 (1). Antiretroviral therapy (ART) is lifesaving for adults and children living with HIV-1, but lifelong due to the persistence of transcriptionally silent, latent integrated proviruses residing in memory CD4+ T cells that are not cleared with current antiretroviral treatment (2–5). As the population of persons living with perinatal HIV-1 ages towards adolescence and young adulthood, the resulting immune activation potential of the viral reservoir and its effects on immune activation and exhaustion marker expression may be distinct. Research efforts to find therapies that restrict and eliminate HIV-1 reservoirs towards ART-free remission in persons living with HIV-1 are underway (6); identifying immune and transcriptomic correlates of HIV-1 reservoir size in longstanding perinatal HIV-1 infection is important towards this goal.

Memory CD4+ T cells constitute the predominant HIV-1 reservoir in adults (7–10). Within the memory CD4+ T cells compartment, HIV-1 persists in different subsets. These include central (Tcm), transitional (Ttm) and effector memory T cells (Tem), for which Tcm is the dominant reservoir (7, 11). In children on long-term ART, Ttm were found to harbor the highest frequency of HIV-1 proviruses, although the extent to which these are replication-competent were not analyzed (11). Naïve CD4^+^ T cells were recently found to also be a reservoir for HIV-1 in adults (12–14), which may be a more abundant in perinatal infection given the dominance of naïve CD4^+^ T cells present in infancy and later childhood (12). Comparative studies of HIV-1 reservoir distribution in various CD4+ T cell subsets in children are limited due to the large blood volumes required to fractionate sufficient CD4+ T cells for study.

Multiple assays are used to measure HIV-1 reservoir size in children and adults living with HIV-1 (15, 16). Total HIV-1 DNA in peripheral blood mononuclear cells (PBMCs) is the most studied (17), with the caveat that total HIV-1 DNA substantially overestimates HIV-1 reservoir size as >90% of the proviruses persisting on ART are defective and therefore non-infectious (18). The newly developed multiplex HIV-1 DNA assay, the Intact Proviral DNA assay (IPDA), allows discrimination between defective and intact proviruses, providing a molecular assay to measure HIV-1 reservoir size (19, 20). This approach is particularly useful for pediatric infections given the relatively low blood volumes required for obtaining sufficient PBMCs for molecular testing. The IPDA, however, does not provide information on the capacity of the proviruses to be expressed, and is also limited by its current optimization for only HIV-1 subtype B infections (19). IPDA specific for non-subtype B infection was recently reported but needs further validation (21). The Tat/Rev Induced Limiting Dilution Assay (TILDA) is also used to quantify HIV-1 reservoir size by assessing transcriptional competence under maximal CD4+ T cell stimulation conditions for multiply-spliced HIV-1 RNA transcripts.

Immune activation and exhaustion markers correlate with HIV-1 reservoir size, as measured by HIV-1 DNA, in adult infections, providing measures of immune correlates of proviral reservoir size (8, 22). Levels of PD-1, TIGIT and LAG-3 on CD4+ T cells were found to correlate with total HIV-1 DNA in adults on long-term ART (8). A recent study in ART-suppressed children (median age of 12.5 years) found a similar correlation between PD-1 and TIGIT and total HIV-1 DNA (23), confirming the potential relevance of these immune exhaustion surface biomarkers as correlates of HIV-1 persistence in both adult and perinatal infections.

We determined the frequencies of immune activation (CD69, CD25, HLA-DR) and exhaustion (PD-1, TIGIT, LAG-3 and TIM-3) markers on different CD4+ T cell subsets (Tn, Tcm, and Ttem) in PBMCs of long-term suppressed children, adolescents and young adults living with perinatal HIV-1, and examined their correlations with HIV-1 reservoir size as determined by the IPDA and the TILDA, and also their correlations with CD4+ T cell transcriptomes. We posit that identifying immune correlates of HIV-1 reservoir size in longstanding perinatal HIV-1 infection, across different ages, may aid with biomarker profiling in evaluating efficacy of HIV-1 remission and cure strategies targeting reservoir elimination.

## 2 Materials and methods

### 2.1 Study population

Ten children, adolescents and young adults living with perinatal HIV-1 infection who participated in our long-term Pediatric Reservoir Cohort study at the Johns Hopkins Pediatric and Adolescent HIV/AIDS Program and the University of Maryland Division of Pediatric Immunology and Adolescent Medicine Program were studied (**Table 1**) (24). The inclusion criteria for study were confirmed perinatal HIV-1 infection and sustained virologic suppression defined as undetectable plasma viral load (<20 copies/mL) at all clinical visits for one or more years. Intermittent viremia with two or fewer consecutive low-level (<400 copies/mL) detectable viral load measures during virologic suppression was allowed.

**Table 1:** Demographic and antiretroviral treatment histories, virologic and immunologic profiles of the study participant.

Participant ID	Age at analysis (yr)	Sex	Race	HIV-1 subtype	ART at analysis	Nadir CD4 Absolute Count	DVS (yr)	Total HIV-1 DNA copies/10^6^ CD4+ T cells	Intact HIV-1 DNA copies/10^6^ CD4+ T cells	3’ Defective HIV-1 DNA copies/10^6^ CD4+ T cells	5’ Defective HIV-1 DNA copies/10^6^ CD4+ T cells	% intact of total HIV-1 DNA
**0113**	15.9	M	AA	B	TAF/FTC/EVG/C	213	4.32^a^	31.44	3.82	22.63	5.00	12.14%
**0117**	17.7	F	Black	C	ABC/3TC/DTG	196˜	8.19	963.28^n^	N/A	N/A	N/A	N/A
**0300**	17.8	F	AA	B	TAF/FTC/BIC	384	15.30	211.50	13.55	77.42	120.52	6.41%
**0301**	22.1	F	AA	B	TAF/FTC/BIC	300	5.86	262.91	13.40	150.34	99.17	5.10%
**0304**	15.8	F	Black	A/G	ABC/3TC/DTG	533˜	12.80	345.26^n^	N/A	N/A	N/A	N/A
**0305**	14.9	F	Asian	A/E	TAF/FTC/RPV	227	9.06	424.40^n^	N/A	N/A	N/A	N/A
**0306**	11.5	F	AA	B	TAF/FTC/BIC	374	4.06	22.76	5.06	7.59	15.17	22.23%
**0307**	10.8	F	White/Mixed	B	TAF/FTC/BIC	1315	4.96	212.36	15.15	171.73	25.48	7.13%
**M0105**	16.8	F	AA	B	ABC/3TC/DTG	740˜	1.36	110.32	7.96	34.75	67.61	7.22%
**M0113**	13.8	M	AA	B	ABC/3TC/DTG	715˜	12.65	52.84	3.64	41.52	11.33	6.89%

DVS, Duration of Virologic Suppression; M, Male; F, Female; AA, African American; TAF, tenofovir alafenamide; FTC, emtricitabine; EVG/C, elvitegravir/cobicistat; ABC, abacavir; 3TC, lamivudine; DTG, dolutegravir; BIC, bictegravir; RPV, rilpivirine. ^a^Suppressed for 12.4 years with brief ART interruption for 4 weeks before resuppression for 4.32 years. ^n^Non-subtype B IPDA intact measurement, only total HIV-1 copies/10^6^ CD4+ T cells are shown. ˜Incomplete patient history, lowest absolute CD4+ on record shown.

### 2.2 Isolation of PBMCs from whole blood

Peripheral blood mononuclear cells (PBMCs) were isolated within 24 hours of collection from whole blood using Ficoll-Paque gradient centrifugation (GE Healthcare, Chicago, IL), cryopreserved and stored in 90% FBS containing 10% DMSO in liquid nitrogen until further use. HIV-1 seronegative donor blood cells obtained from New York Blood Center (New York City, NY) were used as controls to optimize the antibody concentrations for the multiparameter flow cytometry panel (**Supplementary Table 1**).

### 2.3 Flow cytometry analyses of immune activation and exhaustion markers

One million cryopreserved PBMCs were thawed and rested in 4 mL of complete RPMI medium (RPMI 1640 with Glutamax, 10% FBS, 1% penicillin and streptomycin) in a 37°C incubator overnight to restore immunophenotypic markers (25) before staining for flow cytometric analyses. The optimal concentrations of antibodies were determined by serial titrations to determine the concentration that gave the highest stain index. Cells were washed with staining buffer (PBS with 1% FBS) before staining in a 5-ml round bottom tube with each of the following antibodies (**Supplementary Table 1**): T cell surface markers (CD3 APC-R700, CD4 BV711, CD8 BUV496), immune activation markers (CD25 PE-Cy7, CD69 BUV737, HLADR BV786), immune exhaustion markers (TIGIT BUV395, LAG-3 PE, PD-1 BUV661, TIM-3 PE-CF594), memory T cell subsets (CD45RA APC, CCR7 BB700, CD28, BUV805), and CCR5 BV650 (BD Biosciences). Isotype controls were added in corresponding Fluorescent Minus One (FMO) controls at the same concentrations as the corresponding antibodies to adjust for non-specific fluorescence. Cells and antibodies were incubated at room temperature in the dark for 30 minutes before being washed with staining buffer, then fixed and permeabilized with Foxp3/Transcription Factor Staining Buffer Set (eBioscience, San Diego, CA). After the final incubation, cells were washed twice with Permeabilization Buffer and resuspended in 200 µl staining buffer, stored at 4 °C away from light before analysis on a Symphony A3 flow cytometer and FACSDiva version 10 (BD Biosciences, Franklin Lakes, NJ) through the Johns Hopkins Bloomberg School of Public Health Flow Cytometry and Immunology Core. Cell phenotypic data were analyzed using FlowJo (version 10.2, Tree Star). Gating was defined using FMO and isotype controls. Data were collected for total CD8+, CD4+ T cells and subsets: Naïve (CD45RA+ CCR7+ CD28+ CD95-), Central Memory (CD45RA- CCR7+), Transitional Memory (CD45RA- CCR7- CD28+) and Effector Memory (CD45RA- CCR7- CD28-) (**Supplementary Figure 1**). Percentage of cells positive for activation and exhaustion markers were determined within each subpopulation, with gating determined using FMO controls (**Supplementary Figure 2**).

### 2.4 HIV-1 DNA quantification

Three million CD4+ T cells were enriched from PBMCs by negative selection (Miltenyi Biotec, Bergisch Gladbach, Germany). Genomic DNA was isolated from CD4+ T cells using the Gentra Puregene kit (Qiagen, Germantown, MD) according to manufacturer’s directions. HIV-1 DNA was quantified using the IPDA with the QX-200 Droplet Digital PCR system (Bio-rad, Hercules, CA) as previously published (19, 26). HIV-1 DNA quantitation was assessed as intact, 5’ defective or 3’ defective/hypermutated proviruses with primer sequences specific for HIV-1 subtype B (**Supplementary Table 2**) (19). Proviral load was expressed as total, intact, 3’ defective and 5’ defective proviral genomes copies per million CD4+ T cells. For non-subtype B infections, only the total HIV-1 DNA measure was analyzed for correlations, as the assay is not optimized for discriminating intact from defectives in non-subtype B infections (**Table 1**) (19).

### 2.5 Induced HIV-1 reservoir size

Previously reported data on the size of the induced proviral reservoir was determined by a modified TILDA (Enhanced TILDA), specifically optimized for pediatric infections (7) and were used for comparative analysis (24). Briefly, ten million PBMCs were thawed and enriched for CD4+ T cells by negative selection (Miltenyi Biotec, Bergisch Gladbach, Germany). The cells were stimulated with 1µg/mL PHA, 100 ng/mL PMA and 1µg/mL ionomycin for 18 hours in the presence of ARVs as previously published (24). Following stimulation, cells were plated in 22 replicates of the following dilutions: 18000, 9000, 3000 and 1000 cells per well. The presence of induced provirus was detected using reverse transcriptase PCR (RT-PCR) followed by quantitative PCR, with primers matching the individuals’ HIV-1 subtype (**Supplementary Table 3**). The concentration of inducible virus is represented as multiply-spliced RNA producing units per million CD4+ T cells (msRUPM).

### 2.6 Analyses of CD4+ T cell transcriptomes

An aliquot (200,000) of the purified CD4+ T cells that were analyzed for the flow cytometric studies was saved in one ml of TRIZOL solution (Thermo Fisher, Waltham, MA) for RNA-seq analyses after resting overnight. RNA-seq was performed on an Illumina NovaSeq 6000 at the Johns Hopkins Single Cell and Transcriptomics Core. The paired-end sequencing data were aligned to human reference genome hg38 using HISAT2 (27). The normalized gene expression values in terms of transcript per million (TPM) were obtained using StringTie (28).

The data discussed in this publication have been deposited in NCBI’s Gene Expression Omnibus (29) and are accessible through GEO Series accession number GSE209828 (https://www.ncbi.nlm.nih.gov/geo/query/acc.cgi?acc=GSE209828).

### 2.7 Statistical analysis

Statistical analysis was carried out with GraphPad Prism version 8.0 and R version 4.1.3. Multiple comparisons between assay measures were analyzed with a Friedman test and a *post hoc* Dunn’s Multiple Comparison Test, with significance at p < 0.05. Correlations were analyzed using the non-parametric Spearman correlation test with two-tailed p value and considered statistically significant at p < 0.05. To generate the heatmap, the percentage of each cell population was standardized to have zero mean and standard deviation of one across all patients. The heatmap was then generated using the R package ComplexHeatmap (30). The dendrograms on the top and the left side of the heatmap represent the clusters of the participants and the cell types based on hierarchical clustering, respectively. The colors in the heatmap represent the standardized values (i.e., z-scores) of the cell type percentage where red represents a high percentage and blue represents a low percentage.

The correlation among the percentage of cell populations and selected features were visualized using R package corrplot based on Spearman’s correlation (31). The pairs of features that have significant correlation (Spearman’s correlation test p-value < 0.05) were shown using dots where blue color represents a positive correlation and red color represents a negative correlation. For each feature, the p-values were also converted to False Discovery Rate (FDR) using the Benjamini-Hochberg Procedure (**Supplementary Table 4**) (32).

To exclude the effects of age and duration of virologic suppression (DVS) on the comparison between the gene expression and flow cytometry data, age and DVS were regressed out using a linear regression model for each gene and each flow cytometry feature (i.e., percentage of cell population). The Spearman’s correlation was then calculated between each gene and each flow cytometry marker. Genes with absolution correlation larger than 0.75 for at least one flow cytometry feature were retained. K-mean clustering was applied to group these genes into 10 clusters.

### 2.8 Pathway analysis

Ingenuity Pathway Analysis (IPA) spring release 2022 was used to find enriched biological pathways for genes comprising each of the ten identified clusters from RNA-seq data. Pathways that were enriched to an unadjusted p-value <= 0.05 were identified for each cluster.

## 3 Results

**Table 1** summarizes the demographics, ART and virologic profiles for the study participants. The median age at study was 15.8 years (IQR 14.1-17.5; range 10.8-22.1); 80% (*n* = 8) were female, and 80% (*n* = 8) were Black/African; 70% acquired HIV-1 subtype B. The median duration of virologic suppression was 7.0 years (IQR 4.48-11.8; range 4.06-15.3), 90% (*n* = 9) participants were on integrase inhibitors-based ART (**Table 1**).

**Figure 1:**
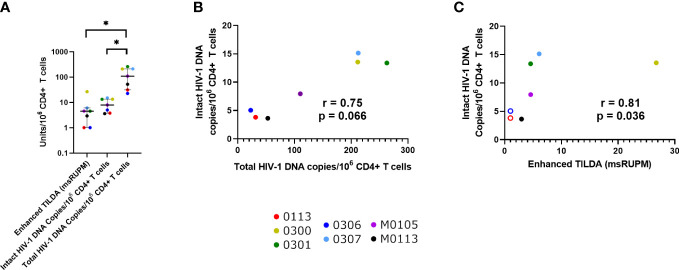
Quantification of the proviral reservoir in participants with HIV-1 subtype B (n = 7/10), using the enhanced TILDA and IPDA. **(A)** The levels of multiply spliced RNA producing units/10^6^ CD4+ T cells(msRUPM), and the levels of intact and total HIV-1 DNA copies/10^6^ CD4+ T cells. Median and IQR are shown. Significance is determined by a Friedman test with Dunn’s multiple comparison test (*p < 0.05). Correlations between Intact HIV-1 DNA copies/10^6^ CD4+ T cells and **(B)** Total HIV-1 copies and **(C)** multiply spliced RNA-producing units/10^6^ CD4+ T cells. Open circles represent undetectable msRUPM.

The median total HIV-1 DNA concentration was 211.90 copies/10^6^ CD4+ T cells (IQR 67.20-324.70) (**Table 1**). When only individuals with HIV-1 subtype B were assessed, the median total HIV-1 proviral load was 110.30, and intact HIV-1 proviral load was 7.96 copies/10^6^ CD4+ T cells (**Figure 1A**), with an estimated 7.13% of total proviruses being intact (IQR 6.41%-12.14%). The median inducible proviral load by Enhanced TILDA was 4.55 msRUPM (IQR 1.01-6.08) (24). There was a positive correlative trend between the intact and total HIV-1 copies/10^6^ CD4+ T cells (p = 0.066) (**Figure 1B**). A positive correlation between the intact HIV-1 copies/10^6^ CD4+ T cells and the Enhanced TILDA msRUPM was found (p = 0.038) (**Figure 1C**).

**Figure 2:**
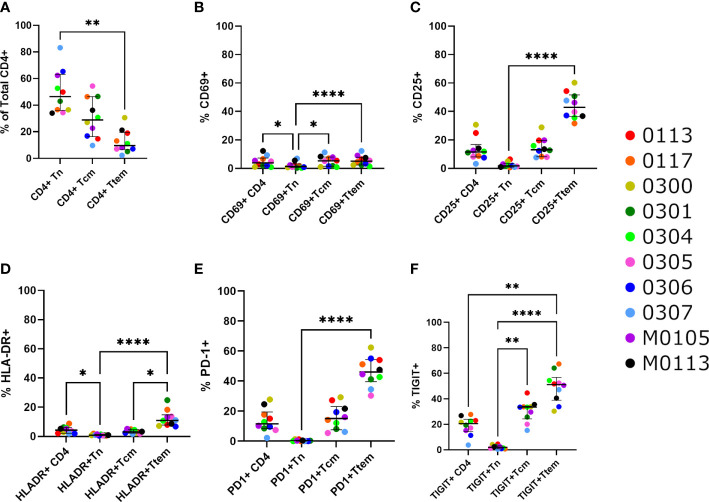
Activation and exhaustion markers on CD4+ T cell subsets. **(A)** Percentage of naïve CD4+ T cells (CD3+CD4+CCR7+CD45RA+CD28+), central memory CD4+ T cells (CD3+CD4+CCR7+CD45RA-CD28+) and a combination of transition (CD3+CD4+CCR7-CD45RA-CD28+) and effector (CD3+CD4+CCR7-CD45RA-CD28-) memory CD4+ T cells within the CD3+CD4+ T cell population. **(B-F)** The percentage of cells with the activation markers **(B)** CD69, **(C)** CD25 and **(D)** HLA-DR, and the exhaustion markers **(E)** PD-1 and **(F)** TIGIT, within CD4+ T cells. Significance is determined by a Friedman test with Dunn’s multiple comparison test (*p < 0.05, **p < 0.01, ***p < 0.001, ****p < 0.0001).

The median frequencies of Tn, Tcm and Ttem CD4+ T cells were 46.4%, 28.9% and 9.74% respectively, with Tn being the most abundant CD4+ T cell subset (**Figure 2A**). Tem comprised a small fraction of the total CD4+ T cell pool at a median of 0.66%. The activation markers (CD69, CD25, HLA-DR) were present at median frequencies of 3.92%, 11.5% and 4.32% of total CD4+ T cells, respectively (**Figures 2B–D**). The immune exhaustion markers PD-1 and TIGIT were present at median levels of 11.6%, 20.6% of total CD4+ T cells, respectively (**Figures 2E, F**). Expression of LAG-3 and TIM-3 were low overall on CD4+ T cells (median 0.23% and 2.03%, **Supplementary Figure 3**), with no clear difference among the different T cell subsets. CCR5 expression was detected at a median of 0.23% on total CD4+ T cells.

We observed significant differences in the presence of CD69, CD25, HLA-DR, PD-1, and TIGIT between CD4+ T cell subsets (**Figures 2B–F**). Overall, Ttem was the most activated and exhausted subset, and there were significantly low but detectable levels of Tn cells expressing immune activation markers. Median levels of CD25 and HLA-DR expressing cells were lowest in naïve CD4+ T cells at 1.98% and 0.83%, respectively, compared with memory subsets (**Figures 2C, D**). There was a higher level of cells expressing HLA-DR in Ttem compared with Tcm CD4+ T cells (median 10.95% vs 3.05%; p=0.0032). The proportion of cells expressing the exhaustion markers PD-1 and TIGIT was observed to increase significantly as cells entered more differentiated states (**Figures 2E, F**). The median levels of PD-1 were 0.20% on Tn, 15.1% on Tcm and 46.0% on Ttem (**Figure 2E**), and the median levels of TIGIT were 1.98% on Tn, 33.5% on Tcm and 51.1% on Ttem.

The proportions of T cells subsets expressing activation and exhaustion markers were also studied in the seven youngest individuals in the cohort, less than 17 years of age (**Supplemental Figure 4**). This sub-analysis represents the children at a median age of 14.9 years (IQR 11.5 – 15.9) who may have a unique and different immunological profile as compared to the three oldest participants. As expected, Tn cells comprised a higher proportion of CD4+ T cells, with a median of 52.8%, as compared to Tcm and Ttem, with medians of 22.7% and 8.28% respectively. Consistent with data from the entire cohort, Ttem had the highest percentage of cells expressing of CD69, CD25, HLA-DR, PD-1 and TIGIT, whilst Tn comprised the lowest percentage of cells expressing these markers by a significant margin (**Supplemental Figures 2B-F**).

**Figure 3:**
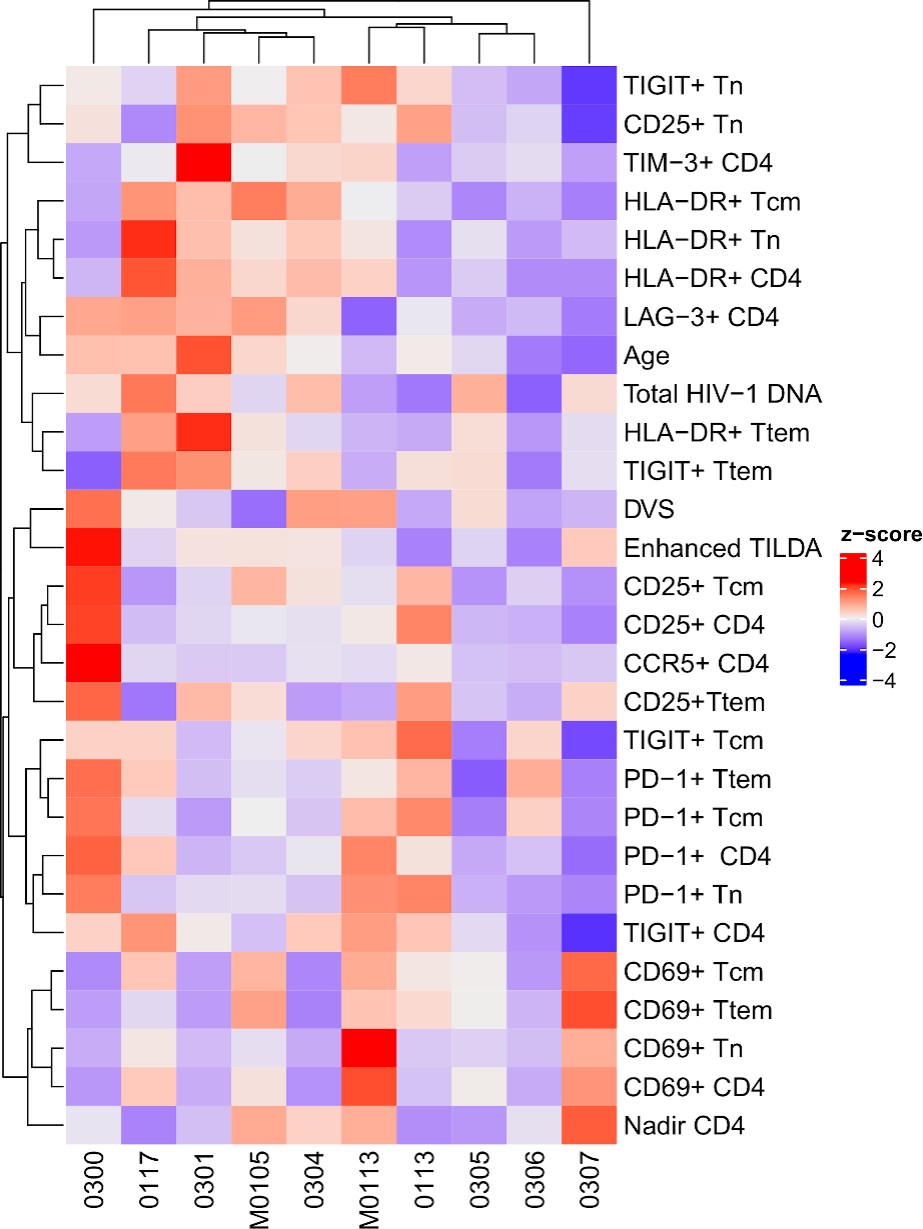
Clustering of the percentage of CD4+ T cells subsets, demographics, total HIV-1 DNA and enhanced TILDA. The heatmap shows the standardized values (z-scores) of each assay measurement across patients. The assays and patients were clustered using hierarchical clustering based on the z-scores. DVS, Duration of virologic suppression.

The relationship among the viral markers studied in the cohort is demonstrated as a heatmap (**Figure 3**). The participants and assay metrics were clustered based on the standardized values (z-score) of each assay, as shown by the accompanying dendrograms. Due to three participants harboring non-subtype B HIV-1, only total HIV-1 DNA, rather than intact proviral load was analyzed. Of note, is the individual participant differences in immune expression profiles. Total HIV-1 DNA was found to cluster most closely with HLA-DR and TIGIT on the Ttem, whereas the Enhanced TILDA clustered with the immune activation markers (CD25 and CD69), and the immune exhaustion markers (TIGIT, PD-1).

**Figure 4:**
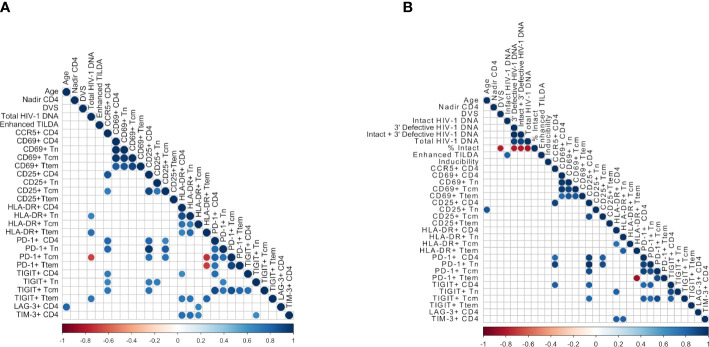
Correlograms depicting significant positive and negative correlations between assay measurements based on Spearman’s correlation. **(A)** Correlations between all samples (n = 10) and **(B)** Correlations between samples from individuals with HIV-1 subtype B (n = 7), including inferred intact provirus by IPDA. Blue color represents a positive correlation and red color represents a negative correlation. Significance was determined using Spearman’s correlation test, at p<0.05.

A correlogram was created to further examine associations between immune activation, exhaustion markers, and measures of HIV-1 reservoir size, as well as CCR5-expression, nadir CD4 T cell counts, age and duration of virologic suppression (DVS) (**Figure 4**). The majority of immune activation and exhaustion markers on the different CD4+ T cell subsets were positively correlated (**Figure 4A**); a positive correlation was found between the proportion of PD-1 and TIGIT expressing CD4+ T cells (r=0.770, p=0.0126), supporting the co-expression of these two exhaustion markers. PD-1 and CD25 expressing CD4+ T cells were also positively correlated (r=0.794, p=0.0088). CCR5 expression on CD4+ T cells was correlated with PD-1 (r=0.794, p=0.009), CD25 (r=0.85, p=0.003) and TIGIT (r=0.709, p=0.027). There was an inverse correlation between Ttem cells expressing HLA-DR and PD-1 expression on Tcm and Ttem. Age was positively correlated with LAG-3 (r=0.8061, p=0.0072). LAG-3 and TIM-3 were both correlated with HLA-DR expressing cells.

**Figure 5:**
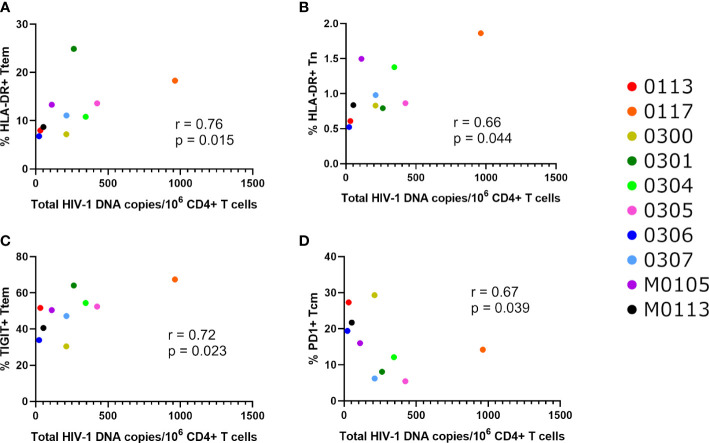
Significant correlations between total HIV-1 DNA and activation or exhaustion markers. Significant correlations between total HIV-1 DNA copies per million CD4+ T cells and the percentage of **(A)** Ttem cells expressing HLA-DR, **(B)** Tn cells expressing HLA-DR, **(C)** Ttem cells expressing TIGIT and **(D)** Tcm cells expressing PD1, as determined using Spearman’s correlation test, at p<0.05.

We also examined correlations between immune markers with the viral assays of reservoir size (Total HIV-1 DNA, intact and defective proviruses, and Enhanced TILDA, **Figures 4**, **5**). Total HIV-1 DNA was significantly correlated with HLA-DR expressing Ttem (r=0.76, p=0.015, **Figure 5A**) and Tn (r=0.66, p=0.044, **Figure 5B**), and TIGIT expressing Ttem (r=0.72, p=0.023, **Figure 5C**). However, total HIV-1 DNA was negatively correlated with PD-1 expressing Tcm (r=-0.67, p=0.039, **Figure 5D**). No correlations were found between immune and exhaustion markers and reservoir size as measured by Enhanced TILDA (**Figure 4A**).

Correlations between the immune activation and exhaustion markers were also examined when restricted to the seven youngest participants (**Supplemental Figure 5**). In this younger cohort, we found the age to be positively correlated with the proportion of CD25+ Tn and Tcm, and with LAG-3+ CD4+ T cells. Interestingly, TIM-3 was inversely correlated with the proportion of CD25+ Ttem, but positively correlated with HLA-DR+ cells. We found similarly that total HIV-1 DNA was positively correlated with HLA-DR+ Ttem and negatively correlated with PD-1+ on Tcm and Ttem. CCR5 was also positively correlated to CD4+ T cells expressing CD25. The Enhanced TILDA was positively correlated with the CD4 nadir, although this analysis is limited due to the number of participants with incomplete clinical histories.

In the seven participants with HIV-1 subtype B infection, we analyzed associations with intact and defective proviral loads (**Figure 4B**, **Supplementary Figure 6**), No correlations were found between intact HIV-1 DNA and immune and activation markers, although this may in part be due to the lower number of participants assessed. Finally, the percentage of intact proviruses detected was inversely correlated with DVS, confirming that intact proviruses decreases over time on ART with longstanding perinatal HIV-1. When CD8+ T cell surface markers were analyzed, no correlations were found between total HIV-1 DNA or Enhanced TILDA and activation or exhaustion markers (data not shown).

**Figure 6:**
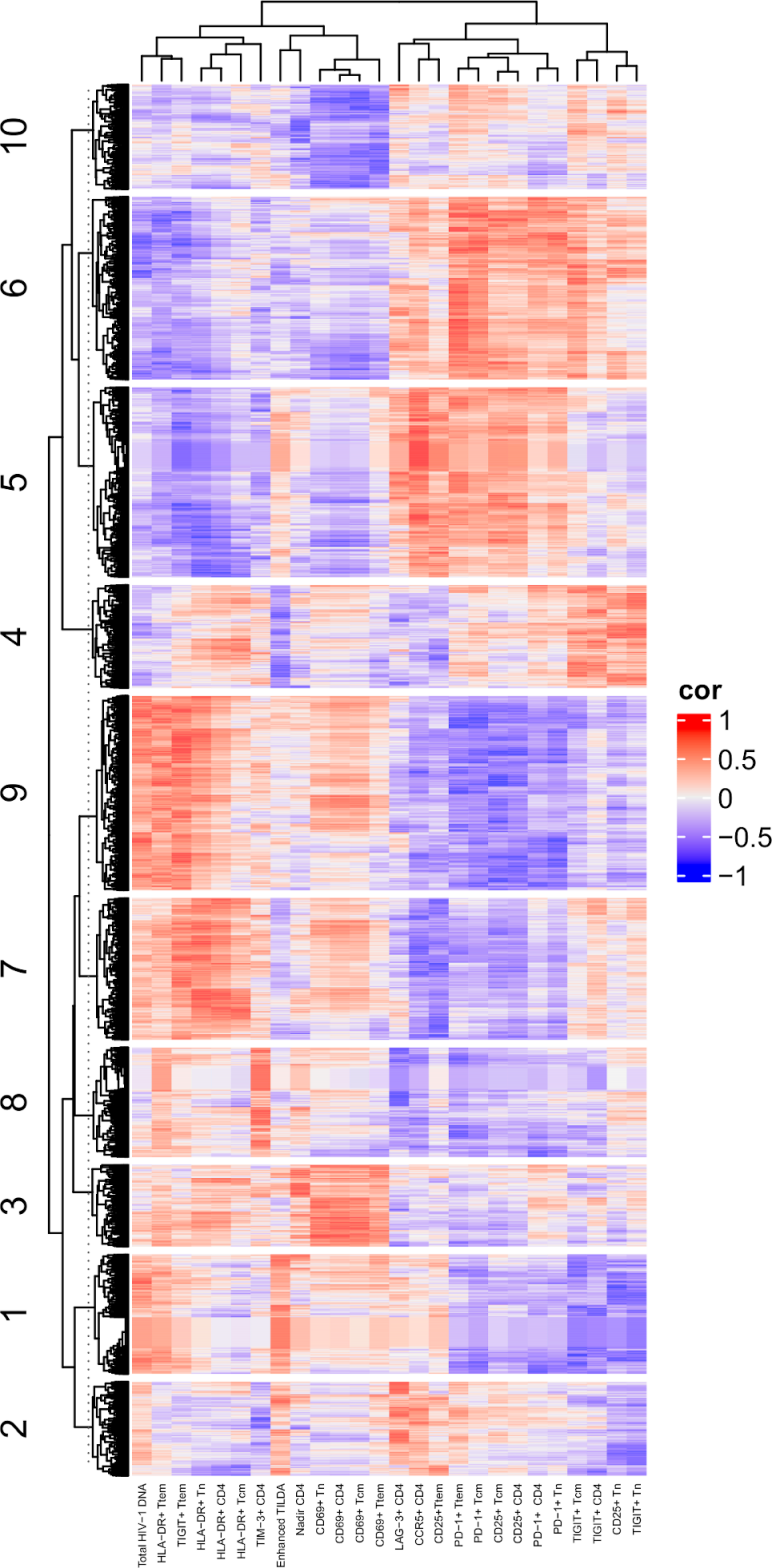
Correlations of assays to RNA-seq. A heatmap showing the Spearman’s correlations between RNA-seq and assay measurements are presented. DVS, Duration of virologic Suppression. In the heatmap, each row represents a gene, and each column represents an assay. Only genes with absolution correlation larger than 0.75 with at least one assay were shown. The genes were grouped into 10 clusters based on K-mean clustering and the cluster IDs were indicated in the heatmap.

To determine associations between cell surface marker expression and cellular transcription, gene expression profiling obtained from RNA-seq was analyzed. Genes were grouped into 10 clusters based on the virologic and immune markers after adjusting for age and DVS (**Figure 6**). Here again, total HIV-1 DNA and cells expressing HLA-DR were closely clustered. As above, measures of reservoir size with the Enhanced TILDA clustered more closely with cells expressing CCR5, PD-1 and CD25. Using Ingenuity Pathway Analysis, the 10 transcriptional clusters can be interrogated for up or downregulation of specific cellular pathways (**Supplementary Table 5**). Of note is cluster 1, in which high levels of total HIV-1 DNA was associated with gene expression profiles linked with immune response and signaling pathways such as PD-1/PD-1L and IL-15. These data confirm that the linkage between cellular transcription and the cell surface marker expression may be influenced by the presence and activity of HIV-1 proviruses.

## 4 Discussion

Most studies of HIV-1 persistence focus on measures of HIV-1-infected cell concentrations, largely using total HIV-1 DNA. Recent developments in viral reservoir assays, including the IPDA and TILDA, have improved the ability to further quantify intact and defective proviruses, and the induced reservoir, especially in the context of therapeutics aimed at ART-free remission and cure (15). This is especially true in infants living with perinatally acquired HIV-1, where early and very early ART causes dramatic reductions in the proviral load and an accurate quantification of the latent reservoir is crucial (33). Few studies have examined immune correlates of HIV-1 persistence in youths and young adults with perinatal HIV-1, although it has previously been shown that soluble immune and activation markers persist in adolescents living with perinatal HIV-1, despite early virologic control (34). It is unknown to what extent immune activation and exhaustion markers on different CD4+ T cell subsets can reflect HIV-1 reservoir size. In this study, we quantified HIV-1 reservoir with the IPDA in a cohort of youths and young adults living with perinatally acquired HIV-1, in whom we previously reported on the inducibility of the latent HIV-1 reservoir with the Enhanced TILDA (24). With multi-parametric flow cytometry, which allowed for simultaneous phenotypic characterization of their immune activation and exhaustion profiles of different CD4+ T cell subsets, along with transcriptomic analyses through RNAseq, we identified unique immune activation and exhaustion marker profiles that are significantly correlated with measures of HIV-1 reservoir size. Additionally, Ingenuity analyses showed potential cellular pathways of interest in ART- suppressed adolescents and young adults living with perinatal HIV-1.

We identified in this cohort that HLA-DR and TIGIT expression on Ttem was the strongest correlate of HIV-1 infected CD4+ T cell frequencies as measured by total HIV-1 DNA. This is in agreement with another study where HLA-DR on Ttm was also shown to be correlated with DNA viral load in adolescents (23). In contrast to studies in adults and one study in children living with HIV-1, where PD-1 expression was correlated with total HIV-1 DNA, we found total HIV-1 DNA to be negatively correlated with PD-1 expressing Tcm. These findings were also supported by the positive correlation between total HIV-1 DNA and HLA-DR transcripts and the inverse correlation between total HIV-1 DNA and PD-1 transcripts as measured by RNA-seq. The inverse correlation between PD-1 and proviral HIV-1 DNA is counter to results in previously published works in both adults and children (8, 23, 35, 36), as well as other work showing correlations between increased frequencies of PD-1+ CD4+ T cells and plasma viral load (37). This lack of correlation may be in part due to the persistence of defective proviruses that can still be expressed, with relevance to the immunopathogenesis of persistent immune activation despite durable ART (10, 38, 39). Interestingly, the exhaustion markers LAG-3 and TIM-3 were positively correlated to T cell subsets expressing HLA-DR, suggesting that the relationship between activation and exhaustion is multi-faceted. No strong correlations were found between immune and exhaustion markers and reservoir size as measured by enhanced TILDA or intact proviral load.

The contribution of HLA-DR+ CD4+ T cells to HIV-1 persistence was highlighted in recent studies in adults showing higher levels of HIV-1 infection in HLA-DR+ cells compared with HLA-DR- memory CD4+ T cells during prolonged ART, along with higher proportions of intact HIV-1 DNA (35, 40). Previously, we reported on a two-fold lower baseline proportion of CD4+ T cells expressing HLA-DR in the same cohort (median 4.55%, IQR 2.56-7.07%) compared to adults living with behaviorally acquired HIV-1 (median, 10.52%; IQR 9.07%-16.99%) at similar HIV-1 DNA levels during suppressive ART. Here we found similar low-levels of HLA-DR+ CD4+ T cell levels by multi-parameter flow with a median of 4.32% (IQR 2.38-5.82%). However, we observed high levels of exhaustion markers, especially PD-1 (median 11.55%; IQR 8.81-17.03%) and TIGIT (median, 20.55%; IQR 15.70-22.88%) on total CD4+ T cells, despite durably suppressive ART, highlighting the immune exhaustion associated with longstanding perinatal infection. These levels were two-fold higher than those reported in adult infection by Fromentin et al. (PD-1+ CD4+ median 4.3%; IQR 2.8-8.0%. TIGIT+ CD4+ median 9.9%; IQR 8.1-12.9%. n=48) (8), where correlations between HIV-1 DNA and PD-1 were observed. The high levels of PD-1 (median 46%; IQR 39.5%-54.3%) and TIGIT (median 51.1; IQR 38.9, 56.8) expressions in Ttem observed in our virally-suppressed pediatric cohort is consistent with another study of ART-treated perinatal infections (age 8-15 yrs. old), when compared to HIV-1 negative youths (37). Together, these findings suggest that even with long-term virologic suppression in perinatal infection, high levels of immune exhaustion exist for which the long-term immune consequences are unknown.

CCR5 expression has been known to regulate the efficiency of HIV-1 pathogenesis during acute infection (41, 42). SIV studies also revealed non-pathogenic infections associated with low CCR5 expression level (43, 44); both studies suggest a role for CCR5 as an indicator of HIV-1 pathogenesis and disease progression. In our study, CCR5+ CD4+ T cells clustered more closely with the inducible reservoir size than total HIV-1 DNA; this relationship was also confirmed by the cellular transcriptomics data. CCR5 expression on CD4+ T cells was also highly correlated with the immune activation marker CD25, and the exhaustion markers PD-1 and TIGIT. Thus, the level of CCR5 expression maybe more strongly modulated by intact, inducible provirus. We also identified that a small subset of naïve CD4+ T cell expressed CCR5, as well as activation and exhaustion markers interrogated, reflecting the potential for naïve CD4+ T cells to be permissive to HIV-1 infection. Indeed, naïve CD4+ T cells have been recently shown to harbor HIV-1 (14, 45).

While most of the study participants are adolescents living with perinatal HIV-1, the cohort nevertheless represents a broad range of ages (10.8 to 22.1 years). In order to determine whether correlations could still be seen in the younger individuals, a sub-analysis was performed of the seven youngest participants. Despite the reduction in statistical power due to the smaller sample size, the positive correlations between total HIV-1 DNA and HLA-DR+ Ttem, and negative correlations HIV-1 DNA and PD-1+ Ttem and Tcm, were still observed. This suggests that these correlations are present in late childhood and adolescence and likely maintained even into young adulthood. We saw an age effect on the expression of LAG-3 (positively correlated with age), revealing immune exhaustion despite longstanding virologic suppression. A negative correlation between the percent of intact proviruses and duration of virologic suppression was seen; the decrease in intact proviruses with longstanding ART-suppressed infection is also observed in adult infection (46), in support of ongoing clearance of intact provirus during durably suppressive ART. Although a correlation between the nadir CD4 and the Enhanced TILDA was observed in this cohort, this analysis is complicated by the incomplete history of a few participants. Thus, the lowest available CD4+ count was employed, which may not reflect the true CD4 count at start of ART.

Our study was limited by sample size with only a small cohort of 10 participants and lack of HIV-1 seronegative controls for the immune activation, exhaustion and transcriptomic analysis, along with T cell subset analyses for total and intact proviruses. A few participants also had incomplete clinical histories, leading to analyses such as correlations with nadir CD4 values and DVS to be made with the information available. In addition, the use of the IPDA to quantify the intact reservoir is not fully validated for non-subtype B infections (19), limiting the analyses of intact proviruses to seven participants. Nevertheless, simultaneous assessment of immune activation and exhaustion markers with multiple reservoir assay measures provides a comprehensive snapshot of the physiologic states of different CD4+ T cell subsets, including naïve CD4+ T cells, in the context of HIV-1 infected cell burden. We identified high levels of PD-1 expression on CD4+ T cells but that HLA-DR and TIGIT on Ttem as a biomarker correlate for infected cell burden in longstanding perinatal infection. Altogether, immune correlates of reservoir size in persons living with perinatal infections across the age-spectrum will require further studies in larger cohorts including those participating in very early and combined immunotherapeutic strategies towards ART-free remission and cure.

## Data availability statement

The datasets presented in this study can be found in online repositories. The names of the repository/repositories and accession number(s) can be found here: https://www.ncbi.nlm.nih.gov/geo/, accession number GSE209828.

## Ethics statement

The studies involving human participants were reviewed and approved by Johns Hopkins Medicine Office of Human Subjects Research institutional review board. Written informed consent to participate in this study was provided by the participants’ legal guardian/next of kin.

## Author contributions

YH, AD, and DP contributed to conception and design of the study. YH, AD, PK, JS, and YC developed and performed the assays. TN aided in panel development, flow cytometry optimization and analysis. WZ, PM, EB, and HJ performed data analysis. AA aided in sample collection and clinical support. YH, AD, and DP wrote the manuscript. All authors contributed to the article and approved the submitted version.
