# Supplementary figures and images for: Immune activation and exhaustion marker expression on T-cell subsets in ART-treated adolescents and young adults with perinatal HIV-1 infection as correlates of viral persistence
Source: Front Immunol. 2023 Mar 23;14:1007626. doi: 10.3389/fimmu.2023.1007626 (PMC10076634; doi:10.3389/fimmu.2023.1007626)

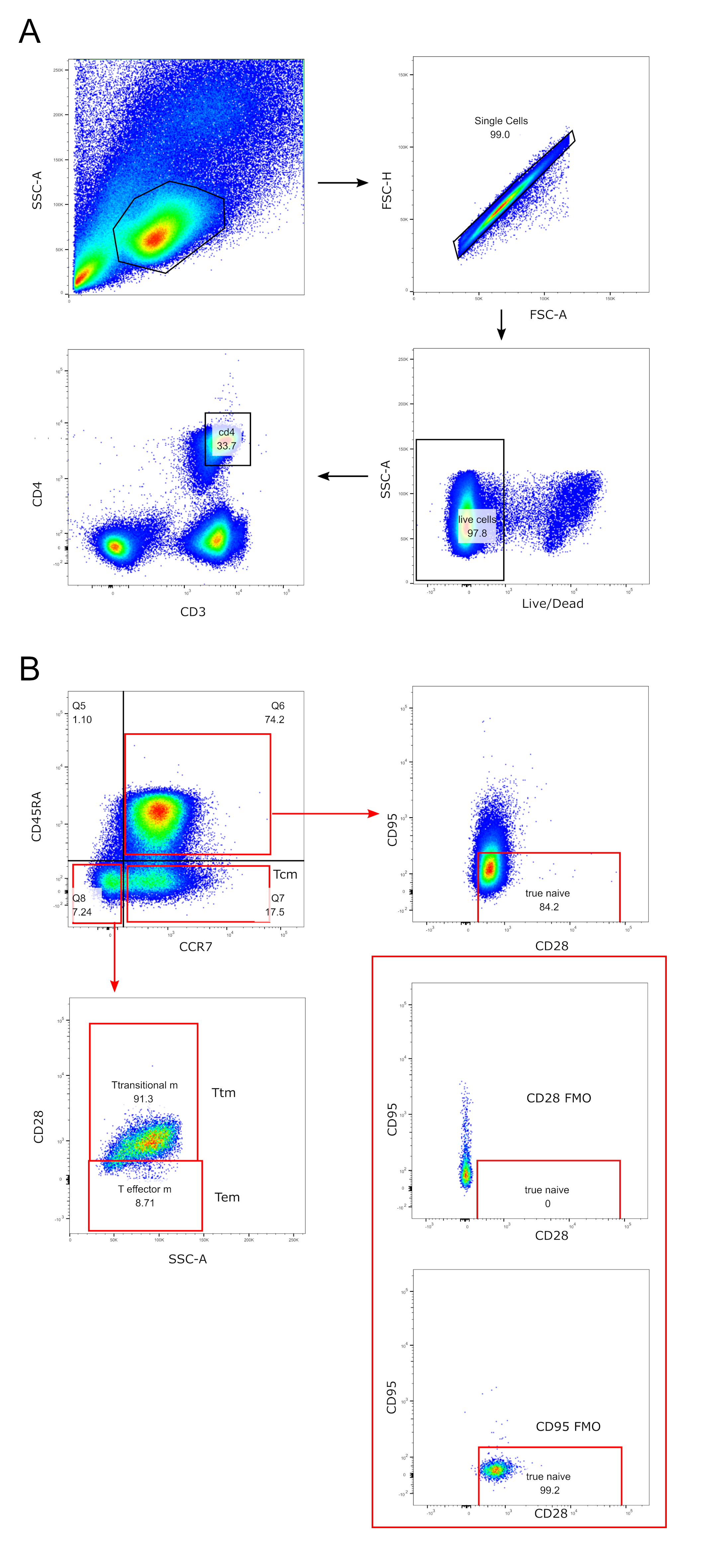

Supplement: Supplementary file 1 [file Image_1.tif]

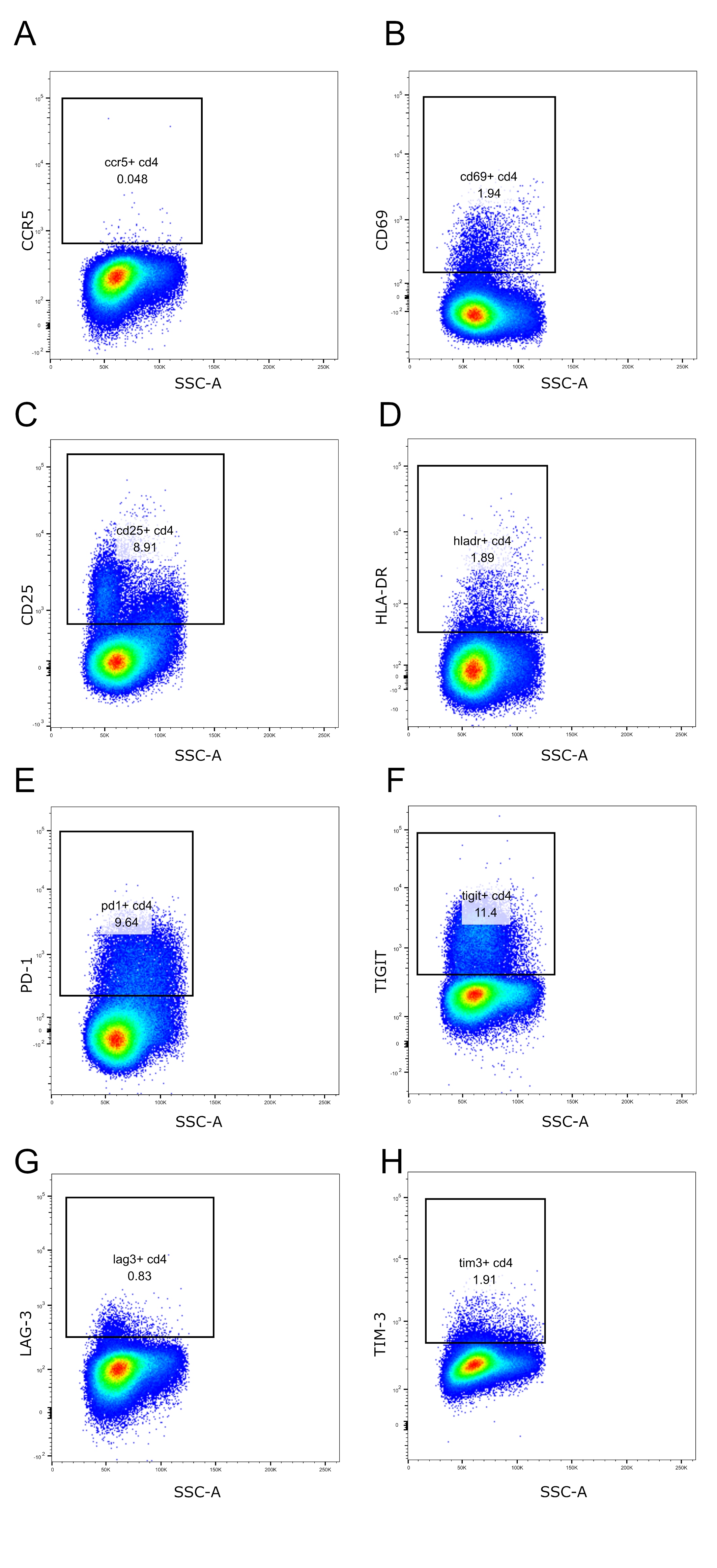

Supplement: Supplementary file 2 [file Image_2.tif]

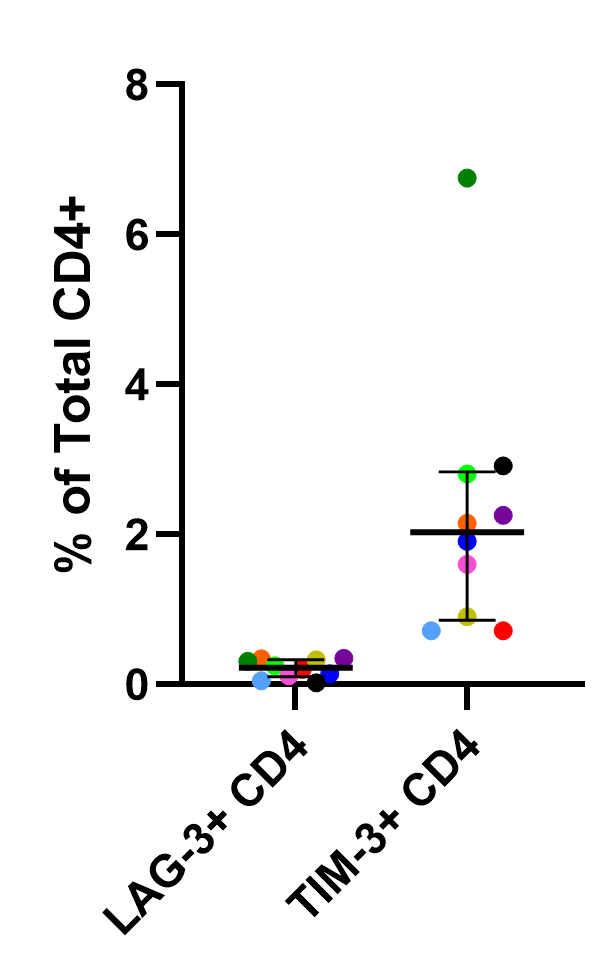

Supplement: Supplementary file 3 [file Image_3.tif]

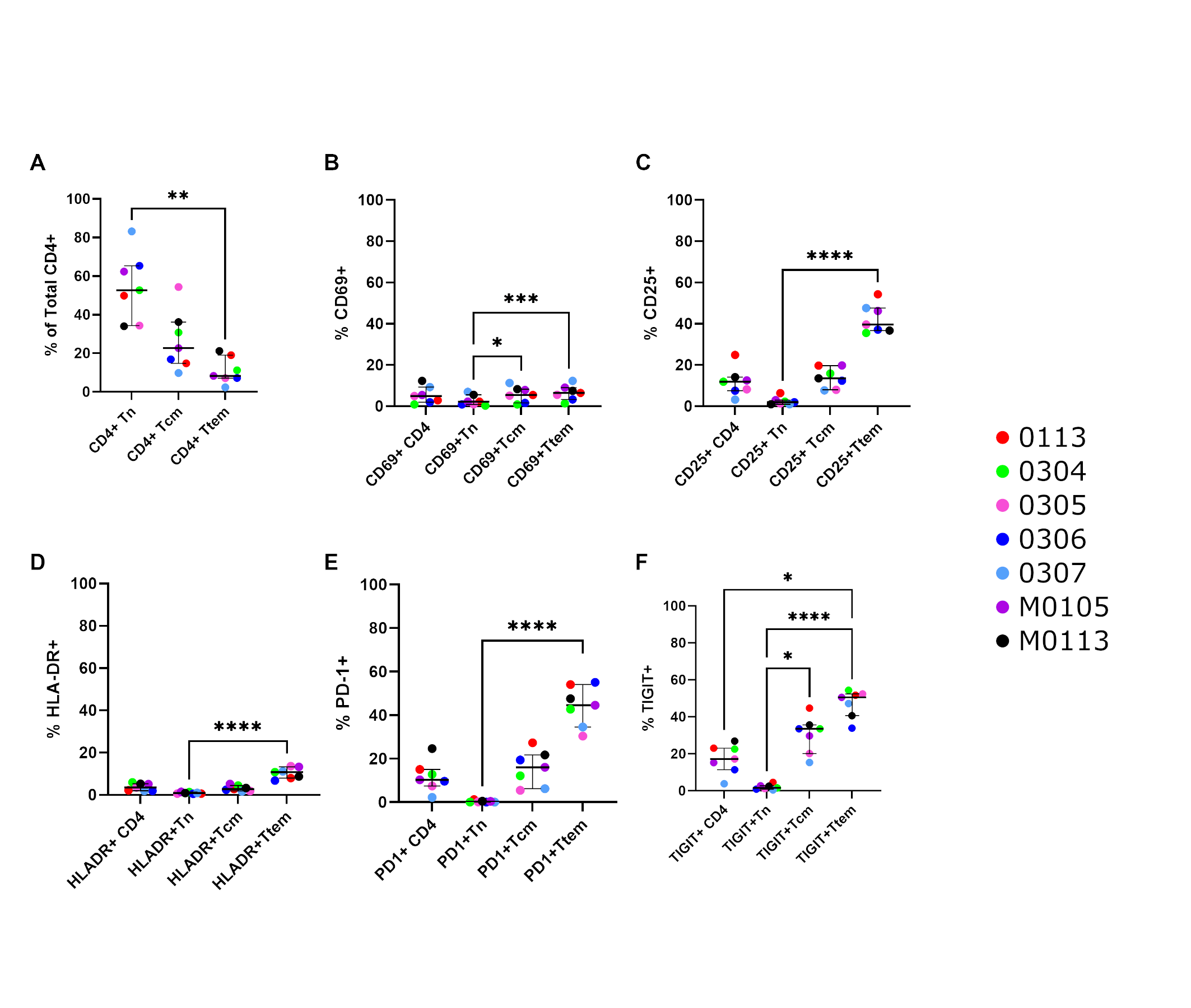

Supplement: Supplementary file 4 [file Image_4.tif]

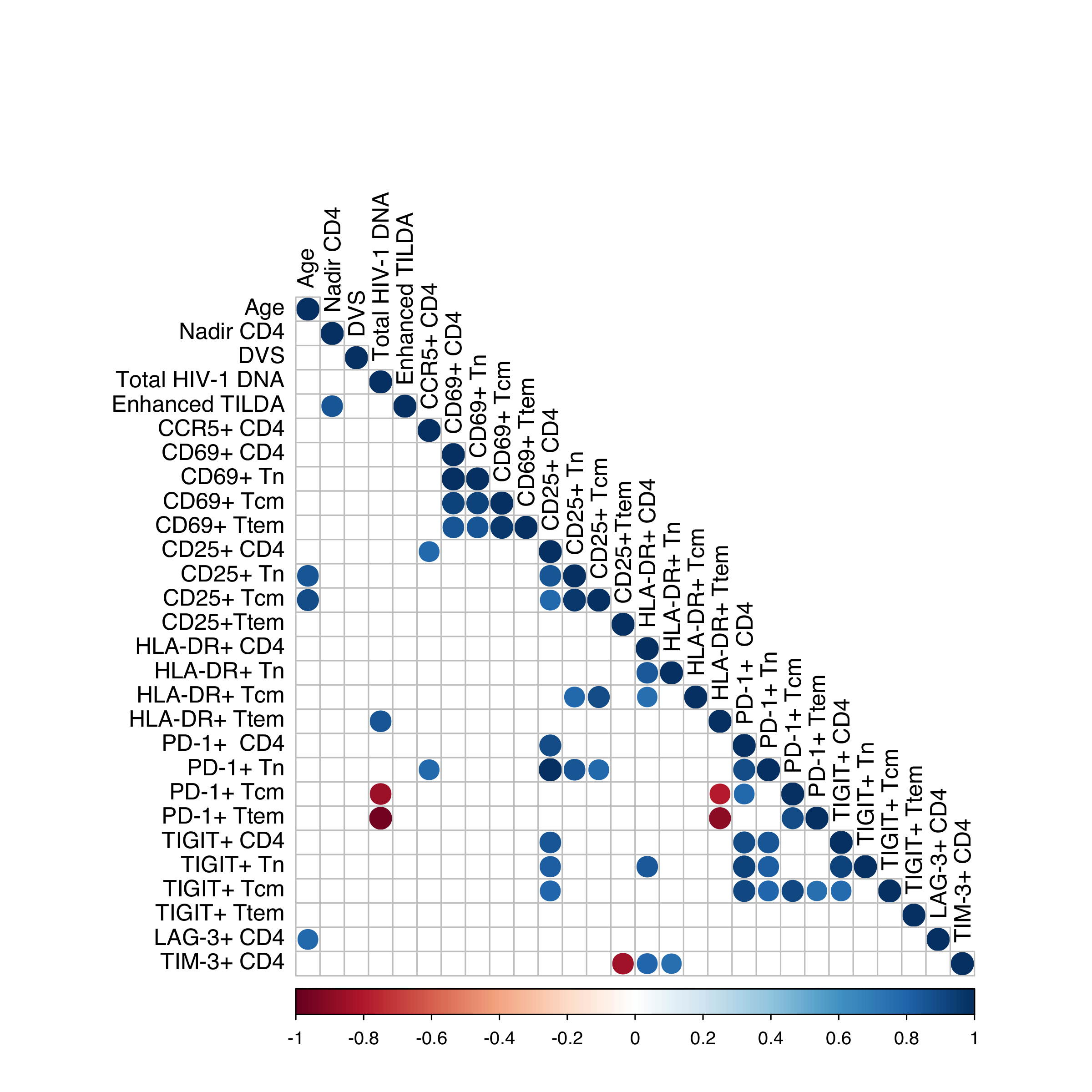

Supplement: Supplementary file 5 [file Image_5.tif]

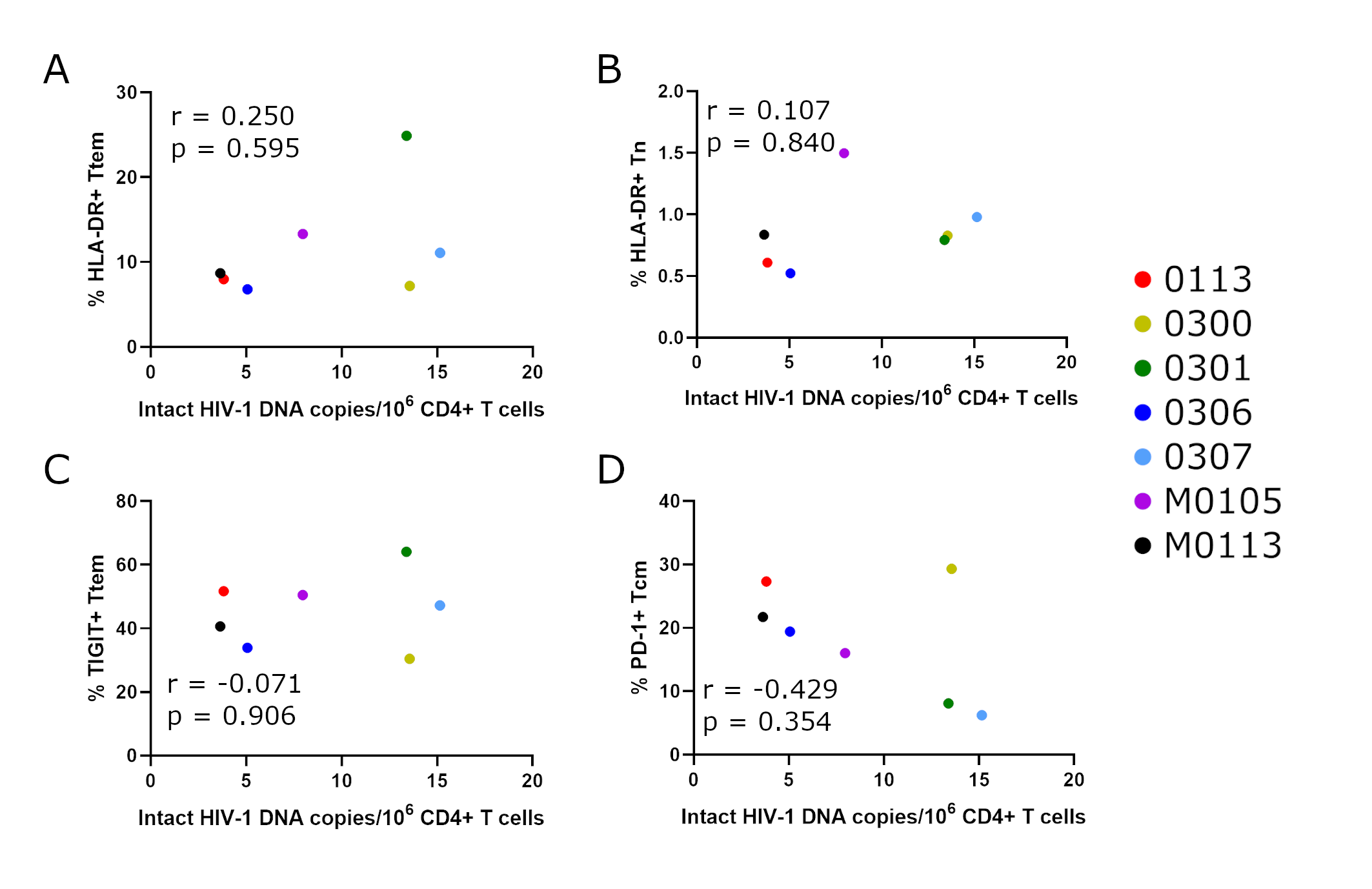

Supplement: Supplementary file 6 [file Image_6.tiff]
