# Supplementary material for: Immune activation and exhaustion marker expression on T-cell subsets in ART-treated adolescents and young adults with perinatal HIV-1 infection as correlates of viral persistence
Source: Front Immunol. 2023 Mar 23;14:1007626. doi: 10.3389/fimmu.2023.1007626 (PMC10076634; doi:10.3389/fimmu.2023.1007626)
Supplement: Supplementary file 7 [file DataSheet_1.docx]

Supplementary Material

# Supplementary Tables

**Supplementary Table 1. Antibodies and isotype controls used for immune-phenotyping**

| Antibody | Fluorophore | Clone | FMO Isotype Control |
| --- | --- | --- | --- |
| CD3 | APC-R700 | UCHT1 | N/A |
| CD4 | BV711 | SK3 | N/A |
| CD8 | BUV496 | RPA-T8 | N/A |
| CD25 | PE-Cy7 | M-A251 | Mouse IgG1 κ, Clone MOPC-21 |
| CD69 | BUV737 | FN50 | Mouse IgG1 κ, Clone X40 |
| HLADR | BV786 | G46-6 | Mouse IgG2a κ, Clone G155-178 |
| CD45RA | APC | HI100 | N/A |
| CCR7 (CD197) | BB700 | 3D12 | N/A |
| CD28 | BUV805 | L293 | N/A |
| CD95 | BV421 | DX2 | Mouse IgG1 κ, Clone X40 |
| CCR5 (CD195) | BV650 | 3A9 | N/A |
| TIGIT | BUV395 | 741182 | Mouse IgG2b κ, Clone 27-35 |
| TIM-3 | PE-CF594 | 7D3 | Mouse IgG1 κ, Clone X40 |
| LAG-3 | PE | T47-530 | Mouse IgG1 κ, Clone MOPC-21 |
| PD-1 | BUV661 | EH12.1 | Mouse IgG1 κ, Clone X40 |
| FVS780 (Viability) | N/A | N/A | N/A |

**Supplementary Table 2. Primers and probe sequences for the IPDA.**

|  | Forward Primer | Reverse Primer | Probe |
| --- | --- | --- | --- |
| Gag | TCTCGACGCAGGACTCG | TACTGACGCTCTCGCACC | /56-FAM/CTCTCTCCT/ZEN/TCTAGCCTC/3IABkFQ/ |
| Env | AGTGGTGCAGAGAGAAAAAAGAGC | GTCTGGCCTGTACCGTCAGC | /5HEX/CCTTGGGTT/ZEN/CTTGGGAGC/3IABkFQ/  /5IABkFQ/CCTTAGG/ZEN/TTCTTAGGAGC/3IABkFQ/ (Hypermutation Probe) |
| RPP30 1 | CCATTTGCTGCTCCTTGGG | CATGCAAAGGAGGAAGCCG | /56-FAM/AAGGAGCAA/ZEN/GGTTCTATTGTAG/3IABkFQ/ |
| RPP30 2 | GATTTGGACCTGCGAGCG | GCGGCTGTCTCCACAAGT | /5HEX/CTGACCTGA/ZEN/AGGCTCT/3IABkFQ/ |

**Supplementary Table 3. Primers and probes for the TILDA based on HIV-1 subtype.**

|  | **Tat1.4** | **Rev** | **Tat2** | **HIVFamzen** |
| --- | --- | --- | --- | --- |
| **B** | TGG CAG GAA GAA GCG GAG A | GGA TCT GTC TCT GTC TCT CTC TCC ACC | ACA GTC AGA CTC ATC AAG TTT CTC TAT CAA AGC A | /56-FAM/TTC CTT CGG /ZEN/GCC TGT CGG GTC CCGTC CC/3IABkFQ/ |
| **A/E** | TGG CAG GAA GAA GCG GAA G | TGT CTC TGY CTT GCT CKC CAC C | GCA GTA AGG ATC ATC AAA ATC CTA TAC CAG AGC A | /56-FAM/TTC YTT CGG/ZEN/GCC TGT CGG GTT CC/3IABkFQ |
| **A/D** | TGG CAG GAA GAA GCG GAG A | TGG TTC TGY CTT GCT CTC CAC C | GCA GTC AGG ATC ATC AAA ATC CTA TAC CAA AGC A | /56-FAM/TTCYTCCGG/ZEN/GCCTGTCGAGATCC/3IABkFQ/ |
| **A/G** | TGG CAG GAA GAA GCG GAG A | TGT CTC TGY CTT GCT CKC CAC C | GCC GTC AGG ATC ATC AAA ATC CTG TAC CAA AGC A | /56-FAM/TTC YTT CGG/ZEN/GCC TGT CGG GTT CC/3IABkFQ |
| **A** | TGG CAG GAA GAA GCG GAR R | GAT CTG YCT CTG YCT TGC TCT CCA CC | GCA GTA AGG ATC ATC AAA ATC CTR TAC CAA AGC A | /56-FAM/TTCTTCCGG/ZEN/GCCTGTCGGGWYCC/3IABkFQ/ |
| **C** | TGG CAG GAA GAA GCG GAG A | GAT CTG YCT CTG YCT TGC TCT CCA CC | GCA GTG AGG ATC ATC AAA ATC YTR TAT CAA AGC A | /56-FAM/TTCYTTCGG/ZEN/GCCTGTCGGGTYCC/3IABkFQ/ |

**Supplementary Table 4. False Discovery Rate of Spearman’s correlations.**

**Supplementary Table 5. Pathway derived from genes with ten distinct RNA-seq clusters using Ingenuity Pathway Analysis**

# Supplementary Figure Legend

**Supplemental Figure 1: Gating of CD4+ T cell subsets from a representative study participant (0306)**. A) Gating for CD4+ T cells: SSC (side scatter) and FSC (forward scatter) were used to identify lymphocytes based on size and granularity. Doublets were excluded to avoid non-specific fluorescence; live cells were picked up based on viability dye expression. Highly fluorescent CD3+ CD4+ cells were identified as target CD4+ T cells. B) Gating for naïve and memory subsets: Tn (naïve T cells) were identified as CD45RA+ CCR7+ CD28+ CD95-, FMOs for CD28 and CD95 are shown; Tcm (central memory T cells) were identified as CD45RA- CCR7+; Ttm (transitional memory T cells) and Tem (effector memory T cells) were identified as CD45RA- CCR7-, CD28 was used to distinguish between the two populations (Ttm: CD28+, Tem: CD28-).

**Supplemental Figure 2: Gating on immune markers on CD4+ T cells from a representative study participant (0306).** Gating against SSC are shown against the following immune markers: A) CCR5, B) CD69, C) CD25, D) HLA-DR, E) PD-1, F) TIGIT, G) LAG-3, and H) TIM-3.

**Supplemental Figure 3:** Percentage of CD4+ T cells expressing LAG-3 and TIM-3. Median and IQR are shown.

**Supplemental Figure 4: Activation and exhaustion markers on CD4+ T cell subsets on young participants (n = 7/10)**. A) Percentage of naïve CD4+ T cells (CD3+CD4+CCR7+CD45RA+CD28+), central memory CD4+ T cells (CD3+CD4+CCR7+CD45RA-CD28+) and a combination of transition (CD3+CD4+CCR7-CD45RA-CD28+) and effector (CD3+CD4+CCR7-CD45RA-CD28-) memory CD4+ T cells within the CD3+CD4+ T cell population. (B-F) The percentage of cells with the activation markers (B) CD69, (C) CD25 and (D) HLA-DR, and the exhaustion markers (E) PD-1 and (F) TIGIT, within CD4+ T cells. Significance is determined by a Friedman test with Dunn’s multiple comparison test, with significance at p < 0.05.

**Supplemental Figure 5: Correlograms depicting significant positive and negative correlations between assay measurements based on Spearman’s correlation, on young participants (n = 7/10).** Blue color represents a positive correlation and red color represents a negative correlation. Significance was determined using Spearman’s correlation test, at p<0.05.

**Supplementary Figure 6: Significant correlations between intact HIV-1 DNA and activation or exhaustion markers.** Only individuals with HIV-1 subtype B are shown. Significant correlations between total DNA copies per million CD4+ T cells and the percentage of (A) Ttem cells expressing HLA-DR, (B) Tn cells expressing HLA-DR, (C) Ttem cells expressing TIGIT+ and (D) Tcm cells expressing PD1, as determined using Spearman’s correlation.
